# Bilingualism for Dementia: Neurological Mechanisms Associated With Functional and Structural Changes in the Brain

**DOI:** 10.3389/fnins.2019.01224

**Published:** 2019-11-14

**Authors:** Sujin Kim, Seong Gak Jeon, Yunkwon Nam, Hyeon soo Kim, Doo-Han Yoo, Minho Moon

**Affiliations:** ^1^Department of Biochemistry, College of Medicine, Konyang University, Daejeon, South Korea; ^2^Department of Occupational Therapy, Konyang University, Daejeon, South Korea

**Keywords:** Alzheimer’s disease, dementia, bilingualism, brain connectivity, cognitive reserve

## Abstract

As the number of older adults increases, the prevalence of dementias, such as Alzheimer’s dementia (AD), vascular dementia, dementia with Lewy bodies, and frontotemporal dementias, also increases. Despite research into pharmacological approaches for treating diverse diseases, there is still no cure. Recently, novel non-pharmacological interventions are attracting attention. Non-pharmacological approaches include cognitive stimulation, alterations in diet, physical activity, and social engagement. Cognitive stimulating activities protect against the negative effects of cognitive decline caused by age-related neurogenerative diseases. Bilingualism is one form of cognitive stimulation that requires multiple aspects of brain activity and has been shown to delay the onset of dementia symptoms in patients by approximately 4–5 years as compared with monolingual patients through cognitive reserve. The purpose of this review was to bilingualism protects against cognitive decline associated with AD and other dementias. We discuss potential underlying neurological mechanisms, including: (1) stimulating adult neurogenesis, (2) enhancing synaptogenesis, (3) strengthening functional connectivity that bilingualism may delay clinical AD symptoms, (4) protecting white matter integrity, and (5) preserving gray matter density.

## Introduction

As the elderly population grows, people around the world must overcome familial, economic, and social challenges in order to protect these individuals against age-related cognitive decline. Of particular importance is disease-related cognitive decline due to Alzheimer’s disease (AD) and other types of dementia that destroy the brain network and consciousness (Antoniou et al., 2013). Many preventative strategies and interventions exist to care for those with disease-related cognitive dysfunction (Livingston et al., 2017). However, numerous drugs have failed to lead to improvements, and there is currently no cure (Mangialasche et al., 2010; Anderson et al., 2017). Thus, alternative, non-pharmacological interventions that may protect cognitive function and delay neurodegeneration in healthy older people have been gaining more attention (Klimova et al., 2017).

Interestingly, older people who engage in brain-stimulating activities, such as reading books and playing board games, are less likely to experience memory loss associated with dementia than those who do not engage in these activities (Akbaraly et al., 2009). Cognitive stimulation strengthens the connections between neurons and promotes healthy cognitive aging (Valenzuela and Sachdev, 2006). Cognitive ability includes memory, pattern recognition, concept formation, attention, perception, action, problem solving, and language (Carpenter, 2013). Similarly, bilingualism evokes brain-stimulation because it requires more neural processing than monolingualism (Marian and Shook, 2012). Moreover, the brain functioning of bilingual people is higher than that of monolingual people, and they generally exhibit better performances across a variety of executive control tasks, including the attention network test (Costa et al., 2008), the Simon task (Bialystok et al., 2004), and the Stroop task (Coderre et al., 2013; Grant et al., 2014) than monolingual people. Additionally, studies have revealed that bilingualism is associated with cognitive advantages throughout the life span (Bialystok et al., 2006; Bialystok and Feng, 2009). Since language learning affects a wide range of brain networks, it may be a favorable solution to promote cognitive reserve. Surprisingly, several studies have demonstrated that the cognitive reserve of bilingual people is enhanced as compared with that of monolingual people, and the onset of AD symptoms in bilingual people are delayed as compared with the onset of AD symptoms in monolingual people (Bialystok et al., 2007; Mortimer, 2014; Perani and Abutalebi, 2015; Woumans et al., 2015; Klimova et al., 2017). Indeed, the increased levels of AD-biomarkers (Aβ and tau) in the cerebrospinal fluid of older adults was alleviated in both early and late bilinguals compared with those in monolinguals, and these effect were superior in early bilingual groups (Estanga et al., 2017).

Despite the accumulation of epidemiological evidence that supports the benefits of language experience on cognitive decline, the underlying neurological mechanisms of these benefits remain unknown. In this review, we suggest potential neurological mechanism by which bilingualism delays dementia along with evidence of clinical and structural changes.

## Hypotheses of the Neurological Mechanisms Underlying Bilingualism

### Adult Neurogenesis

Bilingualism increases the brain activity required to speak two languages (Marian and Shook, 2012; Grady et al., 2015). Sustained exposure to a complicated activity (such as bilingualism) maintains adult neurogenesis at a higher level and improves learning (Kramer et al., 2004). Experience-dependent brain activity provokes the formation of neural connections and structures in order to respond to the demands of managing multiple elements of numerous language systems, such as phonology, semantics, syntax, and grammar (Costa and Sebastian-Galles, 2014). In addition, bilingualism extends to memory tasks (Wodniecka et al., 2010).

There are two neurogenic regions of the adult brain: the subependymal zone of the lateral ventricles and the dentate gyrus (DG) of the hippocampus. The subventricular zone (SVZ) generates the largest number of migratory cells in the adult brain (Goings et al., 2004), and SVZ neuroblasts migrate to the olfactory bulbs (OB) in the adult. Recently, neural stem cells in the adult SVZ have been identified as a potential source of cells for brain restoration (Peterson, 2002). Adult hippocampal neurogenesis occurs throughout life in the subgranular zone of the DG, and evidence suggests that adult-born neurons play a role in brain stimulating activities, such as learning and memory (Kramer et al., 2004; Toda et al., 2019). Adult neurogenesis provokes sustained activity-dependent neural plasticity (Gu et al., 2013), and the relevance of cognitive reserve originates from the prominent role of the hippocampus in higher cognition, such as learning and memory (Kempermann, 2008). Older people who are bilingual perform better on cognitive tasks and have more cognitive reserve than age-matched monolingual people (Bialystok and Feng, 2009). Thus, we provide novel insight that the increasing brain activity through bilingualism may contribute to adult neurogenesis in the brain.

Preclinical studies have reported that granule layer neurons in the DG are produced following brain activity (Hastings and Gould, 1999). Interestingly, unlike other somatic stem cell types, adult neurogenesis is dynamically regulated by activity and experience (Laplagne et al., 2006; Song et al., 2016). Several growth factors are involved in adult neurogenesis, including nerve growth factor (NGF), glial-derived neurotrophic factor (GDNF), and vascular endothelial growth factor (VEGF) (Chen et al., 2005; Frielingsdorf et al., 2007). Cognitive activity may alter the levels of these factors and subsequently change the magnitude of the neurogenic response. VEGF plays a key role in promoting adult neurogenesis (Jin et al., 2002; Licht et al., 2011) by inducing the release of brain-derived neurotropic factor (BDNF) or by acting directly on neuronal precursors through a fetal liver kinase 1 (Flk-1)-dependent mechanism (Schanzer et al., 2004; Nowacka and Obuchowicz, 2013). Additionally, VEGF levels are increased through high intensity hippocampal-dependent cognitive activity (Oh et al., 2012), such as bilingualism. Therefore, bilingualism may contribute to the physiological changes associated with the neurogenesis induced by cognitive activity.

Diseases, such as stroke or ischemia, can induce adult neural progenitor proliferation and the migration of new neurons to sites distal from the injury (Herrera et al., 1999; Parent, 2003; Song et al., 2016). Bilingualism may contribute to cognitive reserve later in life by providing increased neurogenesis and neurons that can travel to relevant circuits. Therefore, the increased brain activity that is associated with bilingualism may be a safeguard against cognitive dysfunction and neuropathology.

### Synaptic Integrity and Synaptogenesis

The brain responds to environmental stimuli, cognitive demand, and behavioral experience by functionally and physically changing in structure (Li et al., 2014). This phenomenon is known as neuroplasticity and has been investigated extensively in many areas. Individuals’ experiences with a second language causes changes in brain structure, and these experience-dependent neural changes can also be affected by the intensity and frequency of second language usage (Bialystok, 2007; Li et al., 2014).

Synaptogenesis, the formation of new synapses, is affected by the ability to speak two languages (Calvo et al., 2015), and a previous study demonstrated that the formation of new synapses underlies learning and memory (Geinisman et al., 2001). Learning has also been shown to result in increased axonal growth of granule neurons and synaptogenesis within the hippocampus (Rusakov et al., 1997; Ramirez-Amaya et al., 2001; Prickaerts et al., 2004). Collections of synapses make defined neural circuits that form networks to perform specific functions (Zhang and Ko, 2018), and synapses produced by experience-dependent activities strengthen neural circuitry (Holtmaat and Svoboda, 2009). Bilingualism recruits alternative brain networks to compensate for those that become damaged during aging and dementia (Marian and Shook, 2012), and the efficient utilization of brain networks to enhance brain function during aging increases cognitive reserve (Schroeder and Marian, 2012).

Experience-dependent alterations modulate BDNF and promote synaptic plasticity (Kang and Schuman, 1995; Figurov et al., 1996; Gold, 2015; Guzman-Velez and Tranel, 2015). GDNF promotes the survival of the dopaminergic frontostriatal circuitry and may be modulated by bilingual experiences (Gold, 2015). VEGF enhances hippocampal-dependent memory by strengthening the neural circuit and increasing neuroplasticity (Cao et al., 2004; Licht et al., 2011) and contributes to cognitive reserve by reducing neuronal loss (Han et al., 2017).

Hence, it could be hypothesized that the bilingual brain responds to multiple language experiences by strengthening synaptogenesis and inducing cognitive reserve. Therefore, we can conclude that language experience can induce structural synaptic plasticity through sufficient brain stimulation and that this is likely to protect against cognitive decline.

### Functional Connectivity

In terms of neural connectivity, bilinguals demand the participation and cooperation of multiple brain areas that are responsible for language processing, including Broca’s area and Wernicke’s area (Hickok and Poeppel, 2007; Li et al., 2015). Moreover, studies have reported that the dorsal lateral prefrontal cortex, dorsal anterior cingulate cortex, and subcortical regions are responsible for language control (Crinion et al., 2006; Green and Abutalebi, 2013; Li et al., 2015). These areas do not function independently but interact with other brain areas involved in language processing (Abutalebi et al., 2009; Price, 2010). This functional connectivity can be confirmed by observing how the responses of two brain regions correlate with each other during neuroimaging procedures (Friston et al., 1993; Biswal et al., 1995). Recent neuroimaging studies revealed that increased functional connectivity is associated with bilingualism (Schlegel et al., 2012; Stein et al., 2012; Chai et al., 2016; Bubbico et al., 2019; Qi et al., 2019).

Schlegel et al. (2012) revealed that the connectivity of the organization of white matter (WM) was increased in English speaking students who were learning Chinese using diffusion tensor imaging (DTI) (Schlegel et al., 2012). Additionally, results from functional magnetic resonance imaging (MRI) of adult English speakers learning French for 12 weeks demonstrated that spontaneous reading and lexical retrieval of the secondary language (L2) was related to an intrinsic functional interaction within the language processing area (Chai et al., 2016). Furthermore, the global cognitive and functional connectivity in the brains of elderly Italian speakers were improved after they completed 4-month-long second language programs (Bubbico et al., 2019).

Changes in the functional connectivity in the impaired network were correlated with improvements in executive function (Kelly and Castellanos, 2014). Executive functions include many higher cognitive activities and regulate inhibitory control and switching processes (Stocco et al., 2012). Inhibitory control plays an important role in determining how to perform successful tasks in various work activities (Dowsett and Livesey, 2000). Bilingual people have advanced inhibitory control because they need to simultaneously regulate the activation of two languages (Bialystok et al., 2004; Stocco et al., 2012). This inhibitory control was reported to activate various brain regions, including the dorsolateral prefrontal cortex, medial prefrontal cortex, inferior frontal gyrus, and basal ganglia (Chambers et al., 2009; Christ et al., 2010). Language switching induced activation patterns in the brains of bilingual speakers (Abutalebi et al., 2008; Garbin et al., 2011; Guo et al., 2011). In addition, results from a quantitative meta-analysis revealed that bilingual language switching significantly activated multiple brain regions, including the midline pre-supplementary areas, left inferior frontal gyrus, left middle frontal gyrus, left middle temporal gyrus, right superior temporal gyrus, right precentral gyrus, and bilateral caudate nuclei (Luk et al., 2011b).

These results demonstrate that learning a foreign-language and bilingualism enhance brain functional connectivity. Increases in the functional connectivity between brain regions that are involved in language processing may result in enhanced executive functions. Furthermore, increased functional connectivity may allow for compensation of age- and neurodegenerative-related cognitive declines. Therefore, strengthening functional connectivity through learning a foreign-language or bilingualism may represent an underlying biological mechanism to delay the onset of AD and other types of dementia.

## Structural Changes in the Brain Due to Bilingualism

### White Matter Integrity

Alterations in neural connectivity are a major pathology of neurodegenerative diseases, especially AD (Palop et al., 2006). The restoration of neural circuits was recently proposed as a strategy for the treatment of AD (Canter et al., 2016). Loss of neural connections is related to widespread network disruption in AD (Daianu et al., 2013), and extensive network deficits cause structural damage to WM (Pievani et al., 2011).

Surprisingly, young bilingual speakers exhibit altered maturation and myelination of WM pathways (Mohades et al., 2015). A study evaluating major WM pathways of elementary school children using magnetic resonance DTI and fractional anisotropy revealed that the mean fractional anisotropy value of the left inferior occipitofrontal fasciculus pathway of bilingual children was significantly enlarged compared with that of monolingual children (Mohades et al., 2015). Using Tract-Based Spatial Statistics analysis, another study showed that the fractional anisotropy values of bilingual people were higher than those of monolingual people in certain WM tracts. Moreover, anatomical brain-imaging studies have demonstrated that adolescents who are bilingual or learning a second language exhibited an increase in WM integrity in the left perisylvian language network (Ferjan Ramirez et al., 2016). These results revealed that learning and using two languages after childhood may have dynamic effects on WM tracts, and this may contribute to maintaining WM integrity later in life (Pliatsikas et al., 2015). Generally, it has been reported that older adults exhibit a decline in WM integrity as result of the gradual process of demyelination (Antoniou et al., 2013). However, bilingual older adults and foreign language learners showed higher WM integrity in the corpus callosum (Luk et al., 2011a, b; Bubbico et al., 2019). In addition, older bilingual speakers show higher WM integrity and stronger anterior/posterior functional connectivity than older monolingual speakers (Luk et al., 2011a).

These results demonstrated that using a second language promotes the integration of global brain areas. Consequently, the slowing down of cognitive functions with age is attenuated in bilingual older adults (Flores and Bronicki, 2017; Bubbico et al., 2019). Additionally, bilingual older adults surpass age-matched monolinguals on executive functioning and attention tasks, such as the Frontal Assessment Battery test (Bubbico et al., 2019), Simon task (Bialystok et al., 2004), and Trail Making Test A-B (Bubbico et al., 2019). These cognitive advantages are associated with neurological correlates, such as maintained WM integrity (Luk et al., 2011a; Antoniou et al., 2013). Bilingualism delays AD symptoms by protecting WM tracks in the frontostriatal and frontoparietal executive control circuitry (Gold, 2015). Thus, superior WM integrity and executive control may act as delaying factors for AD onset through bilingualism-induced cognitive reserve.

### Gray Matter Density

Language learning provides an intensive environmental input into the central nervous system that induces structural changes in the brain (Li et al., 2014) and enhances cognitive reserve. During aging, the volume of gray matter (GM) is reduced in the sensorimotor areas, heteromodal association areas, posterior hippocampus, thalamus, and middle cingulate gyrus. However, the volume of GM declines in the precuneus, parahippocampus, and anterior hippocampal regions during the progression of AD. Both aging and AD decrease GM density in the hippocampus and entorhinal cortex (Raji et al., 2009). Bilingualism increases GM density, improves functional connectivity, and preserves brain structure (Li et al., 2014). Several studies have shown that the density of GM in the anterior cingulate cortex (Abutalebi et al., 2012) and basal ganglia, including the left caudate (Zou et al., 2012) and left putamen (Abutalebi et al., 2013), is increased in bilingual people as compared with that in monolingual people (Perani and Abutalebi, 2015).

Investigations into the structural plasticity of GM in the left inferior parietal region using voxel-based morphometry have shown that GM density was directly proportional to the proficiency of using a second language and inversely proportional to the age at acquisition of a second language (Mechelli et al., 2004). The MRI results of English-German exchange students revealed that the GM in the left inferior frontal gyrus was increased after they studied Germany for 4 years (Stein et al., 2012). Additionally, adult English speakers who studied Chinese for 4 weeks exhibited increased activation in the left superior parietal lobule and left inferior frontal gyrus region (Qi et al., 2019). Another study investigated the effect of early language exposure on Heschl’s gyrus in bilingual and monolingual groups. They found that Heschl’s gyri were larger in bilingual people than those in monolingual people. They also reported that the GM volume of bilingual people was increased as compared with that of monolingual people (Ressel et al., 2012). Furthermore, a previous study indicated that using a second language increases the cortical thickness in related language areas, including the left middle frontal gyrus, inferior frontal gyrus, and superior temporal gyrus, and the volume of the left hippocampus (Martensson et al., 2012; Klein et al., 2014).

These data suggest that this bilingual-associated increase in GM density plays a role in neural reserve and prevention of cognitive decline in AD and aging.

## Limitations and Possibility of Bilingualism for AD Prevention

Although various factors, such as the age and period of secondary language exposure, language proficiency, and usage frequency, are important when evaluating the effectiveness of bilingualism in bilingual individuals, these factors differ from study to study, and some studies do not provide any relevant findings regarding the influence of these factors on bilingualism. These variables make it difficult to integrate and standardize bilingual studies. In addition, social integration and adaptive behaviors may be involved depending on the bilingual learning environment (school, immigration, works, etc.) (Chertkow et al., 2010). Furthermore, the effects of bilingualism can be altered depending on the acquisition order of the mother language (L1) and L2 (Chertkow et al., 2010; Coderre et al., 2013), linguistic similarities between L1 and L2 (Serratrice et al., 2009; Zahodne et al., 2014), and education level of bilingual individuals (Lawton et al., 2015). Moreover, the application of bilingual learning to prevent AD in adulthood involves overcoming multiple hurdles, including motivation, cost, and low frequency of use. Above all, structural and functional changes that occur through learning and acquiring new languages differ between adulthood and childhood. Overall, these points limit the application of bilingualism for the treatment, prevention, or intervention of patients with AD, vascular dementia, dementia with Lewy bodies, and frontotemporal dementias.

However, several studies reported that bilingualism delayed the onset of dementia and also revealed that the age at which a person was exposed to a second language was not limited to adulthood or childhood (Table 1). In addition, bilingualism can induce changes in brain plasticity and functional connectivity if the second language is learned and used throughout the lifetime or if it has only been used for 1–9 months (Schlegel et al., 2012; Chai et al., 2016; Qi et al., 2019). Furthermore, 4 months of learning a second language improved the functional connective and cognitive function in elderly people with normal cognitive function (Bubbico et al., 2019). Additionally, Briellmann et al. (2004) reported that the activation of the whole brain due to bilingualism was not correlated with language learning age, and there was no difference in brain activation between L1 and L2 use in multilingual individuals (Briellmann et al., 2004). Although the application of bilingual learning to interventional or therapeutic purposes for AD and MCI patients is limited, these findings suggest that bilingual learning can increase functional connectivity and cognitive reserve through neurological mechanisms that occur during short- or long-term secondary language exposure in childhood and adulthood (Table 1). In particular, brain alterations in bilinguals, evidenced by radiological images such as CT, MRI, and PET, support these findings (Table 2). Most importantly, studies that have revealed that bilingualism delayed dementia in both childhood and adulthood suggest that bilingual learning may prevent different types of dementia, such as dementia associated with AD.

**TABLE 1:** The studies associating bilingualism to reduced incidence of dementia.

	**The age of L2 exposure**	**Subjects**		**Languages**		**Major findings**		**References**
					
		**Age (Mono/Bi)**	**Number (Mono/Bi)**	**L1**	**L2**			
Neurological mechanisms underlying bilingualism	–	7.2/6.9	20/20	Seven different languages	English	Bilinguals performed better than monolinguals in memory and attention tasks		Bialystok and Feng, 2009
	<10	21.9/21.1	54/55	Nineteen different languages	English			
	<11	70.5 ± 3.0	14/14	English	Different languages	The connectivity of frontoparietal control and default mode networks were increased in bilinguals		Grady et al., 2015
	0	8.5 ± 0.5/8.5 ± 0.5	15/15	English	Thirteen different languages	Bilingual children responded faster in inhibitory control and cognitive flexibility than monolinguals		Bialystok and Viswanathan, 2009
		8.5 ± 0.5/8.6 ± 0.7	15/30	Tamil Telugu	English			
	0	11.1 ± 0.8/11.4 ± 0.9	10/14	French Dutch	Roman German	The MFA value of lIFOF were significantly higher in simultaneous bilinguals than those in monolinguals, and the highest degree change in the MFA value of LIFOF was sequential in bilinguals		Mohades et al., 2015
	3	11.1 ± 0.8/11.3 ± 1.0	10/16					
	<1.5	–/20.3 ± 3.7	–/19	Catalan	Spanish	Language switching revealed activation in the left caudate nucleus, pre-SMA, and ACC in bilinguals. Additionally, the left caudate nucleus was involved in forward switching, and pre-SMA and ACC were involved in backward switching		Garbin et al., 2011
	<5	23.5 ± 3.2/23.5 ± 3.2	–/6	Spanish	English	The dorsolateral prefrontal cortex was involved in language switching. The reaction time in the dorsolateral prefrontal cortex was decreased in bilinguals as compared with that of mixed language individuals. Additionally, the activation of the dorsolateral prefrontal cortex was increased in bilinguals as compared with that in mixed language individuals		Hernandez et al., 2000
	5 ∼ 11^a,b^	23.5 ± 4.5/23.5 ± 4.5	14/14	German	Italian English	The activity of the left putamen was increased in bilinguals when they learned a non-proficient language		Abutalebi et al., 2013
	<5 5 ∼ 13 13<	80.8 ± 4.3/80.8 ± 4.3	18/18	Thirteen different languages	English	Extensive practice controlling both languages helped older adults remember episodic memories		Schroeder and Marian, 2012
	<6	43.0/43.0	10/10	Tamil	English	Bilinguals were more effective at controlling processing than monolinguals. Bilingualism helped delay age-related losses in certain executive processes		Bialystok et al., 2004
		71.9/71.9	10/10					
	<6	25.6/23.9	24/24	Different languages	English	Bilinguals resolved different types of response faster than monolinguals and these patterns increased with age		Bialystok et al., 2006
	<12	66.9/64.5	24/24					
	10.3 ± 7.3	23.0 ± 4.1/21.8 ± 2.4	23/15	English	Chinese	There were no differences in the Stroop interference effect between English speakers who learned Chinese and monolinguals for both languages.	There was no difference in lexical access speed between bilinguals and monolinguals, but the lexical access speed of L2 in bilinguals was delayed as compared with that of L1 in bilinguals	Coderre et al., 2013
	11.0 ± 2.7	23.0 ± 4.1/21.0 ± 1.6	23/22	Chinese	English	The stroop interference effect was reduced for both languages when Chinese speakers learned English as compared with monolinguals		
	11.0 ± 7.0	25.9 ± 6.4/26.8 ± 6.6	66/67	English	Spanish	Selective attention skills of bilinguals were improved at low working memory requirements		Lee Salvatierra and Rosselli, 2010
	19.7 ± 5.7	63.4 ± 8.4/64.8 ± 7.3	42/58					
	12	21.4/21.4	–/24	Chinese	English	The dorsal anterior cingulate cortex and supplementary motor area were involved in local inhibition. The dorsal left frontal gyrus and parietal cortex were involved in global inhibition		Guo et al., 2011
	19	49.0 ± 16.0/49.0 ± 16.0	13/14	Chinese	Sign-language	Switching of sign language and spoken language exhibited high functional activation of the left caudate nucleus region.		Zou et al., 2012
Structural changes in the brain by bilingualism	10.2 ± 4.2	28.2 ± 5.3/31.9 ± 8.1	25/20	Different languages	English	Tract-Based Spatial Statistics analysis indicated that fractional anisotropy values for bilinguals in several WM tracts were higher than those in monolinguals		Pliatsikas et al., 2015
	<11	70.6 ± 3.0/70.4 ± 3.7	14/14	English	Different languages	The temporal lobe cortical thickness was decreased in elderly monolinguals. This was not observed in bilinguals, and the frontal lobe WM integrity was higher in bilinguals than that in monolinguals		Olsen et al., 2015
	<11	70.5 ± 3.0/70.5 ± 3.0	14/14	English	Different languages	The WM integrity in the anterior to posterior functional connectivity was higher in elderly bilinguals than that in elderly monolinguals		Luk et al., 2011a
	5 ∼ 7^a^	26.6 ± 4.2/23.4 ± 4.6	14/17	German	Italian	The activity of GM in the dorsal anterior cingulate cortex was increased in bilinguals		Abutalebi et al., 2012
	<5	-	25/25	English	European languages	The density of GM was increased in early and late bilinguals		Mechelli et al., 2004
	10 ∼ 15		25/33					
	<7	23.1 ± 4.8/21.5 ± 2.7	22/22	Catalan	Spanish	Heschl’s gyri were larger in bilinguals than those in monolinguals		Ressel et al., 2012
	11.6 ± 1.2	25.4 ± 4.3/25.4 ± 4.3	12/12	German	French	The activities of the left caudate and anterior cingulate cortical areas were increased in bilinguals		Abutalebi et al., 2008
	–	6.92 ± 6.80/62.17 ± 5.36	23/23	Chinese	English Cantonese Mandarin	The GM volumes in left temporal pole were increased in the aged bilingual brain		Abutalebi et al., 2014
	–	61.85 ± 6.71/63.2 ± 5.86	30/30	Cantonese	English Mandarin	Bilinguals were increased GM along the ACC		Abutalebi et al., 2015b
	–	71.42 ± 4.88/77.13 ± 4.52	40/45	German Italian	Italian German	The bilingual individuals were increased ECN and DMN metabolic connectivity		Perani et al., 2017

*^*a*^Kindergarten age. ^*b*^Elementary school age. ACC, anterior cingulate cortex; Bi, bilingual; DMN, default mode network; ECN, executive control network; GM, gray matter; lIFOF, left-inferior-occipitofrontal fasciculus; MFA, mean fractional-anisotropy; Mono, monolingual; L1, native language; L2, secondary language; SMA, supplementary motor area; WM, white matter.*

**TABLE 2:** The alterations of radiological imaging in bilingualism.

	**The age of L2 exposure**	**Subjects**		**Languages**		**Major findings**	**References**
					
		**Age (Mono/Bi)**	**Number (Mono/Bi)**	**L1**	**L2**		
Bilingualism related to dementia	4 ∼ 6	78.8 ± 8.0/80.8 ± 6.9	49/37	Welsh	English	Bilinguals outperformed monolinguals in the domain of response conflict and inhibition. There were no differences in executive function tests between monolinguals and bilinguals	Clare et al., 2016
	6 ∼ 18^a^	66.2 ± 26.0/66.2 ± 26.0	257/391	Telugu Hindi	Dakkhini English	The onset of dementia was delayed by 4.5 years in bilingual patients as compared with that in monolinguals	Alladi et al., 2013
	9.3 ± 6.2	76.4 ± 8.5/77.9 ± 7.8	69/65	Dutch	French	Bilingualism delayed AD onset and diagnosis by 4.6 and 4.8 years, respectively	Woumans et al., 2014
				French	Dutch		
	15 ∼ 24^b^	Non-immigrant 76.7 ± 7.8/77.6 ± 7.2	290/89, 19	English	Different languages, French	Bilingualism did not protect against AD, but participants who spoke more than 3 languages were protected against AD	Chertkow et al., 2010
		Immigrant	66/28, 24	French	Different languages, English	Bilinguals showed small protective effects of AD, and more than 3 languages delayed the diagnosis of AD by more than 5 years	
		–	22/135	Different languages	English	Two or more languages delayed the diagnosis of AD by more than 5 years	
	20 ∼ 29^c^	76.5 ± 10/80.8 ± 7.7	109/102	Twenty-one different languages	English	AD diagnoses were delayed by 4.3 years in bilingual patients as compared with those in monolinguals patients	Craik et al., 2010
	20 ∼ 29^c^	66.5 ± 12.3/70.0 ± 10.7	38/36	English	Different languages	The onset of dementia and the first point of clinical visits were later in bilinguals than those in monolinguals	Bialystok et al., 2014
		74.2 ± 11.2/81.4 ± 8.4	35/40				
	20 ∼ 29^c^	71.4 ± 9.6/75.5 ± 8.5	91/93	Twenty-five different languages	English	Bilingualism delayed dementia by 4 years	Bialystok et al., 2007
	20 ∼ 29^c^	74.9 ± 6.9/79.4 ± 6.3	49/19	English	Different languages	In single-domain cases of amnestic mild cognitive impairment, bilinguals were diagnosed later than monolinguals	Ossher et al., 2013
	<6, 6<	52.8 ± 6.4/56.8 ± 6.5 57.6 ± 6.6	100/81, 97	Spanish	Different languages	Bilingualism contributed to cognitive reserve and elevated visual-spatial and executive functions	Estanga et al., 2017
			59/55, 52			Bilingualism alleviated cerebrospinal fluid AD-biomarkers (Aβ and tau)	
	–	80.51 ± 6.50	27/54	Spanish	English	There was no difference in diagnosis proportions for dementia between monolingual and bilingual users	Lawton et al., 2015
		−/72.5 ± 9.4, 74.6 ± 7.8	–/44	Spanish	English	The age of diagnosis of AD delayed with increasing degree of proficiency in each language	Gollan et al., 2011
Cognitive intervention of bilingualism	16 ∼ 18	–	–/10	English	German	The GM in the left inferior frontal gyrus was increased in bilinguals (exchange students; 4 years)	Stein et al., 2012
	20	20.1 ± 1.9/20.1 ± 1.9	27/16	English	Chinese	The connectivity of the organization of WM was increased in bilinguals (nine times per week over 9 month)	Schlegel et al., 2012
	21 ∼ 27	24.9 ± 3.7/24.9 ± 3.7	–/10	English	French	L2 languages were related to an intrinsic functional interaction within the language processing area (French intensive training course; 6 h per day, 5 days per week over 12 weeks)	Chai et al., 2016
	23	23.2 ± 3.7/23.2 ± 3.7	–/24	English	Chinese	Activation in left superior parietal lobule and left inferior frontal gyrus region was increased in bilinguals (3 h per days, 5 days per week over 4 weeks)	Qi et al., 2019
	59 ∼ 79	65.7 ± 3.7/69.5 ± 5.3	12/14	Italian	English	Global cognitive and functional connectivity was improved in the brains of bilinguals (long second language learning program; 2 h per week over 4 months)	Bubbico et al., 2019

*^*a*^School age. ^*b*^Youth. ^*c*^Early adulthood age. Bi, bilingual; Mono, monolingual; L1, native language; L2, secondary language; WM, white matter.*

## Conclusion

**FIGURE 1:**
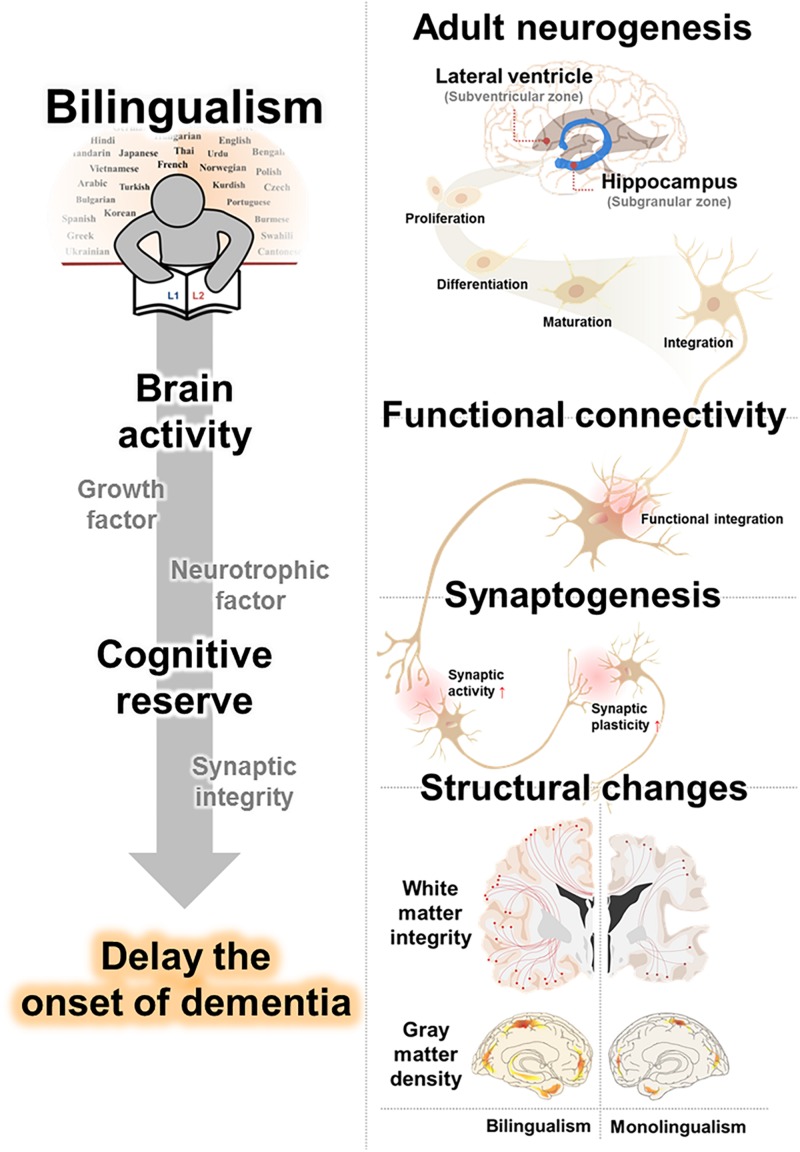
Proposed clausal mechanisms underlying bilingualism-induced delay of dementia. Cognitive reserve from the benefits of language experience on cognitive decline is caused by an increased adult neurogenesis, strengthened synaptogenesis, and enhanced functional connectivity. Bilinguals, accompanied with changes in brain structure, including white matter integrity and gray matter density, delay the onset of dementia.

In this review, we have outlined possible neurological mechanisms that underly the effects of bilingualism on cognitive function and decline. Furthermore, we have integrated studies of dementia delay in bilinguals and summarized evidences for brain alterations in bilingualism. Specifically, we suggested that (1) increased adult neurogenesis, (2) strengthened synaptogenesis, and (3) enhanced functional connectivity may underly the benefits of language experience on cognitive decline. In addition, this review provided evidence for bilingual-induced brain structure conservation, (4) including enhanced WM integrity, and (5) GM density, from age and neurodegenerative related alterations (Figure 1 and Table 3).

**TABLE 3:** The effects of bilingualism: neurological and structural changes in the brain.

**Regions**	**Methods**	**Subjects**		**Alterations**	**References**
				
		**L1**	**L2**		
Frontal region	fMRI	Spanish	English	Left prefrontal activation ↑	Hernandez et al., 2000
	MRI	English	Different languages	Frontal lobe volume ↑	Olsen et al., 2015
	MRI, DTI	English	Different languages	Frontal–occipital and frontal–parietal connectivity ↑	Luk et al., 2011a
	FDG-PET	German	Italian	Dorsolateral prefrontal cortex connectivity ↑	Perani et al., 2017
		Italian	German		
	fMRI	Different languages	Different languages	Broca’s area activation ↑	Kim et al., 1997
Parietal region	MRI	English	European languages	Inferior parietal cortex volume ↑	Mechelli et al., 2004
	MRI	Cantonese	English	Inferior parietal lobule volume ↑	Abutalebi et al., 2015a
			Mandarin		
	FDG-PET	German	Italian	Inferior-, superior- parietal lobules, angular gyrus, posterior cingulum, and precuneus connectivity ↑	Perani et al., 2017
		Italian	German		
Temporal region	sMRI	Chinese	Cantonese	Left temporal pole volume↑	Abutalebi et al., 2014
			Mandarin		
	sMRI	Catalan	Spanish	Heschl’s gyri volume ↑	Ressel et al., 2012
	CT	Different languages	Different languages	Temporal horn volume ↑	Schweizer et al., 2012
Subcortical region	fMRI	German	French	Left caudate nucleus activation ↑	Abutalebi et al., 2008
	PET	French	English	Left putamen activation ↑	Klein et al., 1994
Corpus callosum	sMRI	Cantonese	English	Anterior cingulate cortex volume ↑	Abutalebi et al., 2015b
			Mandarin		
	MRI	Different languages	English	Corpus callosum (genu, body, and splenium) activation ↑	Pliatsikas et al., 2015
	fMRI	German	Italian	Dorsal anterior cingulate cortex density ↑	Abutalebi et al., 2012
	fMRI	German	French	Anterior cingulate cortical areas activation ↑	Abutalebi et al., 2008

*L1, native language; L2, secondary language; CT, computerized tomography; FDG-PET, fluorodeoxyglucose- positron emission tomography, fMRI, functional magnetic resonance imaging; PET, positron emission tomography; sMRI, structural magnetic resonance imaging.*

Such a scientific inquiry would reveal if foreign language learning contributes to cognitive reserve and promotes healthy cognitive aging. However, bilingualism studies are difficult to standardize and change depending on variables, including like that the learning environment, order of acquisition of L1 and L2, and the linguistic similarities between L1 and L2. Nevertheless, bilingualism delays brain damage caused by AD and other dementias in both childhood and adulthood and indicated the potential for cognitive intervention (Table 1). In addition, the substantial brain structures and activation regions also altered in bilinguals (Table 3). Therefore, bilingualism may be considered as part of cognitive multiple interventions for patients with dementia. In conclusion, bilingualism may be a precautionary measure that can be used to has a potential role in delaying the onset and progression of neurodegenerative dementia, including dementia associated with AD.

## Author Contributions

All authors had full access to all the data in the study, took responsibility for the integrity of the data and accuracy of the analysis, contributed to the manuscript revision, and read and approved the submitted version. MM and D-HY conceived the study and acquired the funding. SK, HK, and YN performed the methodology. HK, SJ, and YN investigated the study. SJ, D-HY, and YN provided the resources. SK and HK wrote the original draft of the manuscript. SK, SJ, and YN wrote, reviewed, and edited the manuscript. SK and SJ visualized the study. MM supervised the study.

## Conflict of Interest

The authors declare that the research was conducted in the absence of any commercial or financial relationships that could be construed as a potential conflict of interest.
